# Sports Performance and Injury Epidemiology in Portuguese XV Rugby Union’s 2023 World Cup Preparation in a High-Altitude Center: A Cross-Sectional Study

**DOI:** 10.3390/jfmk10030332

**Published:** 2025-08-28

**Authors:** Carlos Braga, António Cruz-Ferreira, Luiz Miguel Santiago

**Affiliations:** 1 USF Norton de Matos, ULS Coimbra, 3030-790 Coimbra, Portugal; 2 Federação Portuguesa de Rugby, 1600-131 Lisboa, Portugal; antonio.ferreira@ipdj.pt; 3 Faculty of Medicine, University of Coimbra, 3000-548 Coimbra, Portugal; lsantiago@uc.pt; 4 Centre for Health Studies and Research, University of Coimbra, 3004-512 Coimbra, Portugal; 5 Departamento de Medicina, Instituto do Desporto e Juventude, 1250-190 Lisboa, Portugal

**Keywords:** injuries, GPS data, Rugby Union, performance, sports medicine

## Abstract

Background: Rugby Union’s physical demands lead to high injury rates, requiring players to optimize their abilities. Altitude training enhances performance but poses risks to injuries. Methods: This cross-sectional observational study compares the Portuguese Rugby team’s injury rates and Global Positioning System (GPS) performance data during the Rugby World Cup (RWC) 2023 preparation phase. Two medical doctors from the medical Portuguese department diagnosed and recorded all the injuries occurred. GPS players data recorded the following: running distance (RD), high-speed running distance (HSRD), number of accelerations of high intensity (HI), maximum velocity (MV), and percentage of personal maximum velocity (% MV). Data were analyzed by position and growth rate (∆) comparing sea level (Cycle 1) and altitude (Cycle 2). The players were analyzed by injury severity, type, anatomical location, and GPS performance metrics. Results: A higher number of injuries was recorded in Cycle 2 compared to Cycle 1 (∆ = +5 for forwards; ∆ = +3.5 for backs). While average values for MV and % MV showed a downward trend, RD, HSRD, and HI exhibited upward trends. However, none of these differences reached statistical significance. Conclusions: Injury counts and training volume indicators showed upward trends, while MV and % MV declined, though none reached statistical significance. These patterns should be interpreted cautiously, and further research is needed to explore GPS metrics in injury monitoring.

## 1. Introduction

Rugby Union is a global high intensity contact sport characterized by frequent collisions (scrums, maul, rucks), rapid changes in direction, and repeated bursts of sprinting [1]. These physical features make Rugby Union one of the sports with higher rates of injury [2,3]. To perform effectively, rugby players must develop a wide range of physical qualities, including speed, strength, power, endurance, and acceleration capabilities [1].

Currently, with high levels of competition and increasingly shorter recovery times, different training methods have been sought that enhance performance while minimizing injury risk [4,5]. Coaches and medical teams must balance performance gains with athlete well-being, particularly as chronic fatigue and overtraining can negatively impact both [6].

This study aims to compare injury patterns and GPS-based performance trends between two training environments (sea level and high altitude), during the Rugby World Cup 2023 preparation phase of the Portuguese National Rugby XV Team, with analyses conducted according to player position (Forwards and Backs).

Monitoring athlete workload in training sessions or matches through objective data has become increasingly important in this context. Technologies such as GPS tracking now allow a monitoring of external load parameters like running distance, high-speed efforts, and sprint performance [7,8]. Recent technological and scientific progress has made it possible to check the health condition and performance more accurately in a less invasive way [9]. However, the volume of available data can be difficult to manage and interpret to the staff, raising the need for clear, purposeful application in clinical and coaching decisions [9].

Overtraining remains a diagnostic challenge in sports medicine due to the lack of standardized thresholds or criteria to determine when an athlete is exceeding physiological limits [10]. Complementary data such as wellness scores, recovery markers, and training load may help flag early signs of maladaptation.

To help the players to have a better performance it is important to design a pre-season or pre-tournament phase knowing the needs, limitations, and goals of the team players to adapt the training sessions. Playing position may influence injury susceptibility and performance profiles. For instance, forwards and backs are involved in unique movement patterns, collision exposure, and physical demands [11]. Besides that, injury types or locations are more associated with a matchday environment than a training session because of the intensity of the competition [12].

Since the 1990s, altitude training has been widely used to enhance athletic performance at sea level. Literature describes improvements in hematological capacity, as well as central (e.g., ventilation, hemodynamics, neural adaptation) and peripheral (e.g., muscle buffering capacity) physiological factors [13,14,15,16]. However, altitude training also imposes greater cardiorespiratory demands, which may contribute to increased fatigue and elevate the risk of injury [13]. From its early adoption, this dual effect showed the importance of monitoring and individualize training strategies in such environments [17].

## 2. Materials and Methods

A cross-sectional study design was used to assess the growth rate of injury and performance data recorded during the preparation phase of the Rugby World Cup 2023 for an Elite Team, Portugal’s National XV Rugby Union. The preparation phase was completed with two training campaigns at sea level in Lisbon and Quarteira from 26/06/2023 to 19/08/2023 (Cycle 1) and another one carried out at Font-Romeu’s National Altitude Training Center at 1850 m of altitude from 20/08/2023 to 03/09/2023 (Cycle 2). This study was conducted in accordance with the ethical standards outlined in the Declaration of Helsinki. Ethical approval was obtained from the “*Comissão de Ética da Faculdade de Medicina da Universidade de Coimbra*”, under approval number refª CE-031/2024. Informed written consent, was obtained from all participants before data collection. Participants were assured of confidentiality, and all data were anonymized to protect their identities. No invasive procedures were involved, and participation was voluntary, with the right to withdraw at any time without consequences. The participants included all the 33 players from Portugal’s XV Rugby squad, who were called up for the final phase of the RWC who provided informed consent.

Two medical doctors from the medical department diagnosed and recorded all the injuries that occurred, specifying the type, location, and severity. The data was also recorded from the beginning to the end of the injury. The type and location of injury were categorized according to the global consensus guidelines on injury definitions and data collection methods used in Rugby Union injury studies. The severity of the injury was classified as “minor” if the player was able to enter in the next training session or as “major” if the player was unable to join the next training session.

GPS-derived performance data included total running distance (RD, measured in meters), high-speed running distance (HSRD, measured in meters covered at speeds above 18 km/h) [18], and high intensity accelerations (HI, defined as accelerations exceeding 2.5 m/s^2^) [19]. Maximum velocity (MV) was recorded in km/h, and the percentage of maximum velocity (% MV) referred to the proportion of total running performed at the athlete’s individual top speed. For statistical analysis, all GPS variables were categorized by player position. Players were grouped as forwards (props, hookers, locks, flankers, number eight) and backs (scrum half, fly half, centers, wings, fullback), following standard practice in rugby research. Training structure was identical, but drills reflected positional roles: forwards focused more on set pieces, backs on open-field play. The scrum half was classified as a back for consistency with the literature. The GPS model used was *Catapult Vector S7* and it was used in a vest under jersey of each player in every session. GPS monitoring commenced at the start of the main training session, immediately after the warm-up, to specifically capture the primary training load. Distances covered during the warm-up phase were therefore not included in the recorded data.

To avoid confusion in the terms “field position” and “team position”, we prefer to use the nomenclature of “team position”, which refers to the tactical position they occupy and not the position in which they are located on the field of play. Backs and forwards were exposed to same type of training sessions. For both absolute counts (“n”) and percentages (“%”), growth dynamics (∆) was calculated as follows: ∆ = (Cycle 2 − Cycle 1)/Cycle 1.

Descriptive statistics were performed as well as trends were calculated, with the 26th SPSS version. The relationship between GPS-derived parameters and the number of injuries was assessed using the Mann–Whitney U test, comparing delta (∆) values between positional groups (forwards vs. backs), with statistical significance set at *p* < 0.05. This non-parametric approach was chosen due to non-normal data distribution but does not account for the repeated-measures structure of the dataset, which is acknowledged as a study limitation.

## 3. Results

In total, 165 GPS-tracked training sessions were analyzed in Cycle 1 and 164 in Cycle 2, reflecting session-level data across all 33 players.

In Cycle 1, there was a total of 10 injuries (5 minor and 5 major) and in the Cycle 2, a total of 32 injuries occurred (24 minor and 8 major). The number of total injuries in Cycle 1 was higher in forwards (n = 6) and in Cycle 2 was higher in backs (n = 20) (Table 1).

Throughout the preparation phase, there was an increase in the number of injuries, especially in minor injuries, presenting a ∆ = +5.0 increase in the number of minor injuries in forwards and ∆ = +3.5 in backs. The major injuries delta has a lower expression comparing Cycle 1 to Cycle 2 (Table 1).

Regarding the injuries anatomical location in the Cycle 1, there was a higher number of cases in forwards’ feet and toes, while in backs the injuries occurred more in the thigh region. This trend continued during the Cycle 2, with an increase of ∆ = +1.5 in injuries to the anterior thigh and ∆ = +2.5 to the posterior thigh (Table 2).

Considering the type of injury in the Cycle 1, the tendinopathies affected more the forwards (N = 5) while the muscle tears/strains had a higher proportion in backs (n = 4). In Cycle 2, the trend continued in both positions, forwards tendinopathies increased with a ∆ = +0.60 and muscle strain/tear injuries in backs presenting a ∆ = +2.0 increase, representing 17% of all injuries occurred in this cycle (Table 3).

There was a decrease from Cycle 1 to Cycle 2 in the percentage of maximum speed in sprints, in both positions (Table 4 and Table 5).

Evaluating the average running distance covered in meters, during training sessions and matches, there was a ∆ = +0.23 for forwards and ∆ = +0 17 for backs from Cycle 1 to Cycle 2, also increasing the maximum value in both field positions exceeding 5000 m (Table 5 and Table 6).

The high-speed running distance had a positive trend from the first to the second training cycle, in the case of forwards ∆ = +0.11 and in the case of backs ∆ = +0.15. The maximum values remained the same in the forwards while in the backs there was an increase of ∆ = +0.21 (Table 5 and Table 6).

There was a very similar increase in the number of high intensity accelerations over time in forwards and backs (∆ = +0.31 and ∆ = +0.29, respectively). The maximum value of this category had a positive trend of ∆ = +0.53 in forwards and ∆ = +0.47 in backs (Table 5 and Table 6).

The maximum speed reached by the forwards was 29.8 km/h and by the backs was 31.6 km/h (Table 5 and Table 6).

Although there was a positive trend in the RD, HSRD, and HI, and a negative trend in maximum velocity, there was no GPS data value that showed a statistically significant relationship between the variation from Cycle 1 to Cycle 2 (Table 6).

## 4. Discussion

Rugby coaches, strength and conditioning specialists, and support staff aim to ensure players are physically prepared, maintain high performance, and avoid injury throughout the season. Therefore, identifying reliable, evidence-based parameters to monitor player performance and well-being is essential. Total running distance is commonly used to assess training volume, while average speed is often used as a measure of intensity [20].

Forwards are typically involved in high-impact actions (e.g., rucks, scrums, mauls) that demand greater body mass and strength, which may provide some protection against contact-related injuries. [3,21,22]

In contrast, backs cover more open-field distances (30–50 m) at high speeds and rely heavily on agility and acceleration. Consequently, they may be more prone to high-speed tackle-related injuries. [3,22,23].

In this study, descriptive trends suggested that injury frequency was higher in Cycle 2, particularly among backs. This may reflect cumulative fatigue over the 3-month World Cup preparation period, contributing to a higher incidence of minor injuries [2].

The number of major injuries remained stable between cycles, suggesting no clear temporal trend. While accumulated fatigue may raise injury risk overall [2], major injuries in rugby typically result from acute trauma or high-impact mechanisms [2,23,24] and are less influenced by recovery time. [23]. Minor injuries, if not properly managed, can worsen or lead to major injuries, reinforcing the importance of careful return-to-play decisions. [11]

Injury locations in rugby can be influenced by the mechanism of injury and team position [25]. Backs showed more lower-limb injuries, likely due to their greater number of accelerations and decelerations. The thigh, heavily involved in sprinting and braking, was particularly affected [13,22,26]. Running remains the most frequently reported injury mechanism in rugby, which may help explain the higher incidence of thigh injuries among backs in this study [27,28]. This information is very important for the staff team to develop strategies that increase lower-limb resistance.

The emergence of leg injuries in Cycle 2, absent in Cycle 1, coincided with lower-limb loading during this period. These injuries occurred in a closer period to the Rugby World Cup, when intensity of training was sequentially higher, presenting a matchday-like training environment. The literature suggests that while backs may experience fewer injuries overall, their injuries tend to be more severe [3]. Reducing training intensity may decrease collision risk. Sessions focused on technical skills with lower high-impact load may better prepare players while reducing injury risk [29,30]. So, it could be considered to have training sessions more focused on greater repetitions but with lower high-kinetic movements.

Forwards were more affected by tendinopathies linked to repetitive loading, while backs sustained more muscle strains and tears, consistent with fatigue-related injury patterns [2,31]. Both injury types increased from Cycle 1 to Cycle 2, but given the unequal exposure, these patterns cannot be interpreted as true increases in incidence. Soreness is a relevant factor in managing training intensity. Players experiencing soreness or self-perceived fatigue are more prone to injury, making effort monitoring essential [32]. This can be achieved through wellness questionnaires or biological markers (e.g., CK, CRP, IL-6, PTX-3, LDH), although none are definitive [33]. MRI may detect muscle soreness but is often impractical due to costs [33]. A limitation of this study is the absence of direct measures of fatigue, recovery, pain, or muscle damage (e.g., creatine kinase, C-reactive protein, wellness questionnaires, or sleep quality metrics). As such, any references to cumulative fatigue based solely on indirect performance trends. Future studies should incorporate objective and subjective fatigue markers to improve the validity of fatigue-related interpretations.

Between cycles, average running distance increased while maximum speed and % MV decreased. These patterns, although not statistically significant, may reflect rising fatigue, which could have influenced the injury trends observed in Cycle 2 [7].

Although training volume increased (as shown by total distance), peak running speeds declined, possibly due to cumulative fatigue and the higher number of minor injuries [8]. Minor injuries can reduce training quality, affecting the player performance without fully excluding players from sessions.

The highest distances covered occurred in Cycle 2, suggesting increased physical demands. The rise in high-speed running and accelerations may indicate improved stamina [15], but without direct fatigue markers, this should be interpreted cautiously.

Some potential confounders such as session content, baseline fitness, and psychosocial stress may have influenced injury risk and performance but were not controlled for in this study and should be considered when interpreting the findings. The lack of complementary psychological (e.g., wellness questionnaires, perceived stress) data limits the ability to fully interpret the multifactorial causes of injury. Future research should combine GPS-derived metrics with these measures to provide a more holistic assessment of injury risk factors.

One notable limitation is the unequal duration between cycles (Cycle 1: 54 days; Cycle 2: 14 days), which limits direct comparisons in injury frequency or workload. As we lacked exact exposure data (e.g., athlete-hours), training time, detailed training content, and recovery/rest management, only absolute injury counts are presented. Consequently, the higher number of injuries observed in Cycle 2 may be attributable to differences in exposure duration or training density rather than an inherent increase in injury risk. Future research should incorporate precise training load and exposure metrics to enable normalized, rate-based comparisons. A further statistical limitation is that the analysis of delta (∆) values between positional groups was performed using the Mann–Whitney U test, which does not account for the clustering of repeated observations from the same players across sessions. This approach ignores potential intra-player and intra-session correlations, which could lead to underestimated *p*-values. Future studies should consider using mixed-effects models or generalized estimating equations (GEEs) incorporating player-level random effects and relevant covariates (e.g., period, time trend, position, and session content), as well as reporting effect sizes and 95% confidence intervals for greater robustness.

The cross-sectional nature of this study precludes establishing causal relationships between training environment, GPS-derived performance indicators, and injury occurrence. Although differences were observed between cycles, these represent associations within specific time frames rather than longitudinal cause–effect links. Future studies employing prospective cohort or repeated-measures designs across extended periods are required to better understand temporal relationships and causal mechanisms.

This study highlights the potential of GPS data in monitoring athlete readiness and injury risk. Further research could explore the relationship between GPS trends and injury onset or subjective wellness. A major concern is that findings like these may not be adopted in practice by coaching staff. Applying such data in training design could enhance performance and reduce injury risk.

## 5. Conclusions

This study highlights the differences in injury risk and GPS performance trends between rugby field positions during an elite pre-tournament training phase. These findings support the importance of position-specific monitoring and training design to help reduce injury risk and optimize performance. The observed reduction in average maximum velocity and % MV from Cycle 1 to Cycle 2 may signal early signs of fatigue, suggesting that such GPS-derived indicators could serve as practical tools for medical and performance staff. While this study did not include direct fatigue markers or compare injured and non-injured groups, future research should aim to incorporate biological or imaging data and individualized GPS baselines to improve early detection of overload. Overall, these insights contribute to the ongoing effort to refine injury prevention strategies and enhance player management in elite rugby contexts.

## Figures and Tables

**Table 1:** Injury severity.

Injury Severity	Cycle 1						Cycle 2						Delta					
	Forwards		Backs		Total		Forwards		Backs		Total		Forwards		Backs		Total	
	n	%	n	%	n	%	n	%	n	%	n	%	n	%	n	%	n	%
Without Injury	89	93.7	66	94.3	155	93.9	82	87.2	50	71.4	132	80.5	−0.08	−0.07	−0.24	−0.24	−0.15	−0.14
Minor injury	1	1.1	4	5.7	5	3	6	6.4	18	25.7	24	14.6	+5.00	+4.82	+3.50	+3.51	+3.80	+3.87
Major Injury	5	5.3	0	0	5	3	6	6.4	2	2.9	8	4.9	+0.20	+0.21	-	-	+0.60	+0.63
Total	95	100	70	100	165	100	94	100	70	100	164	100	−0.01	0	0	0	−0.01	0

Cycle 1: CAR-Jamor Camp + Browns Sports Resort Camp; Cycle 2: Font-Romeu’s National Altitude Training; n: number of injuries.

**Table 2:** Injury location.

Injury Location	Cycle 1						Cycle 2						Delta					
	Forwards		Backs		Total		Forwards		Backs		Total		Forwards		Backs		Total	
	n	%	n	%	n	%	n	%	n	%	n	%	n	%	n	%	n	%
Head/Face	1	1.1	0	0	1	0.6	-	-	-	-	-	-	-	-	-	-	-	-
Hip/Groin	-	-	-	-	-	-	4	4.3	0	0	4	2.4	-	-	-	-	-	-
Anterior Thigh	0	0	2	2.9	2	1.2	0	0	5	7.1	5	3	-	-	+1.5	+1.4	+1.5	+1.5
Posterior Thigh	0	0	2	2.9	2	1.2	0	0	7	10	7	4.3	-	-	+2.5	+2.4	+2.5	+2.6
Feet/Toes	5	5.3	0	0	5	3	4	4.3	4	5.7	8	4.9	−0.2	−0.19	-	-	+0.6	+0.6
Leg	-	-	-	-	-	-	4	4.3	0	0	4	2.4	-	-	-	-	-	-
Shoulder	-	-	-	-	-	-	0	0	2	2.9	2	1.2	-	-	-	-	-	-
Infectious Disease	-	-	-	-	-	-	0	0	2	2.9	2	1.2	-	-	-	-	-	-
Total	95	100	70	100	165	100	94	100	70	100	164	100	−0.01	0	0	0	−0.01	0

**Table 3:** Injury type.

Injury Type	Cycle 1						Cycle 2						Delta					
	Forwards		Backs		Total		Forwards		Backs		Total		Forwards		Backs		Total	
	n	%	n	%	n	%	n	%	n	%	n	%	n	%	n	%	n	%
Soreness	1	1.1	0	0	1	0.6	4	4.3	2	2.9	6	3.7	+3.00	+2.91	-	-	+5.00	+5.17
Muscle Tear/Muscle Strain	0	0	4	5.7	4	2.4	0	0	12	17.1	12	7.3	-	-	+2.00	+2.00	+2.00	+2.04
Tendinopathy	5	5.3	0	0	5	3	8	8.5	4	5.7	12	7.3	+0.60	+0.60	-	-	+1.4	+1.43
Infectious Disease	-	-	-	-	-	-	0	0	2	2.9	2	1.2	-	-	-	-	-	-
Total	95	100	70	100	165	100	94	100	70	100	164	100	−0.01	0	0	0	−0.01	0

**Table 4:** GPS indicators (Cycle 1).

GPS Indicators	Cycle 1									
	Forwards					Backs				
	Mean	±SD	Median	Min	Max	Mean	±SD	Median	Min	Max
RD (m)	1975.0	972.1	2047.9	348.6	3910.7	2573.9	971.4	2789.5	952.7	4144.8
HSRD (m)	325.2	331.3	220.1	0.0	1340.0	667.1	438.3	548.68	8.5	1545.8
No. accels HI	24.1	14.0	23.0	1.0	60.0	35.6	18.01	34	3.0	71.0
MV (km/h)	23.3	3.0	23.8	16.3	29.8	26.8	2.042	26.833	18.7	31.5
% MV	83.9	8.3	84.9	63.0	99.9	82.4	6.792	83.031	58.0	96.3

RD: running distance; HSRD: high-speed running distance; No. accels HI: number of accelerations of high intensity; MV: maximum velocity.

**Table 5:** GPS indicators (Cycle 2).

GPS Indicators	Cycle 2									
	Forwards					Backs				
	Mean	±SD	Median	Min	Max	Mean	±SD	Median	Min	Max
RD (m)	2419.0	1385.1	2350.4	0.0	5001.2	2998.4	3242.8	1589.0	0.0	5655.0
HSRD (m)	360.3	366.6	203.7	0.0	1342.8	768.6	780.2	558.7	0.0	1871.2
No. accels HI	31.6	21.2	28.0	0.0	92.0	46.0	52.0	32.0	0.0	104.0
MV (km/h)	22.9	4.5	23.0	0.1	29.8	25.8	26.1	4.2	0.4	31.6
% MV	81.9	15.2	84.3	0.4	100.2	79.2	81.4	13.4	1.2	95.1

**Table 6:** GPS indicator deltas.

GPS Indicators	Delta								
	Forwards				Backs				
	Mean	Median	Min	Max	Mean	Median	Min	Max	*p*
RD (m)	+0.23	+0.15	−1.00	+0.28	+0.16	−0.43	−1.00	+0.36	0.240
HSRD (m)	+0.11	−0.07	-	0	+0.15	+0.02	−1.00	+0.21	0.815
No. accels HI	+0.31	+0.21	−1.00	+0.53	+0.29	−0.06	−1.00	+0.47	0.142
MV (km/h)	−0.02	−0.03	−0.99	0	−0.04	−0.84	−0.98	0	0.174
% MV	−0.02	+0.01	−0.99	0	−0.04	−0.84	−0.98	−0.01	0.679

RD: running distance; HSRD: high-speed running distance; No. accels HI: number of accelerations of high intensity; MV: maximum velocity.

## Data Availability

The data presented in this study are available on request from the corresponding author due to privacy and ethical restrictions.

## Abbreviations

The following abbreviations are used in this manuscript:

% MV	Percentage of personal maximum velocity
CK	Creatine Kinase
CRP	C-reactive protein
GPS	Global Positioning System
HI	Number of accelerations of high intensity
HSRD	High-Speed Running Distance
IL-6	Interleukin-6
LDH	Lactate Dehydrogenase
MRI	Magnetic Resonance Imaging
MV	Maximum Velocity
PTX-3	Pentraxin-3
RD	Running Distance
RWC	Rugby World Cup
